# Measuring What Counts to Advance Indigenous Self-Determination: A Case Study of the Nisg̱a’a Lisims Government’s Quality of Life Framework and Survey

**DOI:** 10.1007/s42413-020-00088-1

**Published:** 2020-10-21

**Authors:** Karen Bouchard, Adam Perry, Shannon West-Johnson, Thierry Rodon, Michelle Vanchu-Orosco

**Affiliations:** 1 Université Laval, Québec, Canada; 2 Nisga’a Lisims Government, Quality of Life Department, Terrace, British Columbia Canada; 3 Greater Victoria Coalition to End Homelessness, Director of Research and Data Analysis, Victoria, BC Canada

**Keywords:** Indigenous quality of life survey, Data governance, Self-government, Modern treaties, Canada

## Abstract

Modern Treaties are presented as a means for improving the lives of First Nations, Inuit, and Métis peoples in Canada by providing specific rights, and negotiated benefits. However, the positive impacts of Modern Treaties on Indigenous well-being are contested (Borrows and Coyle 2017; Coulthard 2014; Guimond et al. 2013; Miller 2009; Poelzer and Coates 2015). Developing a more transparent, consistent, collaborative and contextual way of measuring well-being relevant to the cultural realities of Modern Treaty beneficiaries is an important step for generating comparative methods that could systematically demonstrate whether, and under what conditions, such agreements can effectively reduce socio-economic disparities and improve the quality of life of Indigenous communities. The authors first examine previous attempts at measuring Indigenous well-being, then reflect on well-being in relation to the Modern Treaty context. Subsequently, the authors provide an example from one Self-Governing Indigenous Government, the Nisga’a Lisims Government, to collect well-being data through the Nisga’a Nation Household Survey using a mixed quantitative-qualitative method developed through a culturally grounded and participatory approach.

## Introduction

In Canada, Modern Treaties^1^ (otherwise known as Comprehensive Land Claims Agreements or CLCAs) are tripartite agreements between Canada, an Indigenous Nation or group, and a province or territory, designed to ensure the political and legal recognition of Indigenous rights and titles.^2^ These negotiated agreements are protected under section 35 of the *Constitution Act, 1982*, and often held as markers of governmental recognition of, and commitment to Aboriginal rights.^3^ As such, Modern Treaties are expected to strengthen the participation of Indigenous citizens in the Canadian federation, and encourage strong and self-reliant communities across Canada (Canada Minister of the Department of Indian Affairs and Northern Development 2011). Establishing Modern Treaties is equally about relationship-building and reconciliation through the establishment of legally founded and mutually binding obligations that foster a positive long-term relationship between Indigenous and non-Indigenous people.^4^

Academics have been critically engaging with Modern Treaties to study their impacts on income, land, and resource governance with mixed results (Abele and Prince 2006; Alcantara 2017; Alcantara and Davidson 2015; Alcantara and Nelles 2014; Aragón 2015b; Cameron 2019; Papillon 2008; Pendakur and Pendakur 2018; Rodon 2018). Some comprehensive land claim agreements, both with and without associated self-government agreements, have been linked to increases in income and wages for Indigenous households (Aragón 2015a; Pendakur and Pendakur 2018). However, in a study on the effects of the James Bay and North Quebec Agreement, Papillon (2008) found that while Modern Treaties “do not change the socioeconomic conditions and overall well-being of communities” these agreements could enable Indigenous peoples to establish “a governance relationship that better reflects their social, economic and political aspirations” ( 2008:5). A study conducted by Aboriginal Affairs and Northern Development Canada (AANDC, now Crown-Indigenous Relations and Northern Affairs Canada or CIRNAC) with the Inuvialuit Regional Corporation in 2013 echoed the conclusions drawn from Papillon (2008). While Modern Treaties introduced mechanisms that support economic development, Papillon and AANDC found little evidence of improvements to the living conditions of Indigenous communities. The inadequate implementation of Modern Treaty obligations appeared to compromise the ability of Indigenous signatories to fully benefit from the socio-economic opportunities the agreements were intended to provide (Canada Evaluation, Performance Measurement, and Review Branch of Aboriginal Affairs and Northern Development 2013). No methods have yet been developed to comprehensively evaluate and report on how Modern Treaties may reduce socio-economic disparities and enhance the living conditions of Indigenous peoples across Canada. In their Provisional Annual Report, CIRNAC indicated that more comprehensive outcome-based data was required to fully grasp the socio-economic impacts of modern treaties on Indigenous populations and all Canadians (CIRNAC 2019: 2).

In this paper, the authors take a critical look at past efforts by the Canadian Government to measure Indigenous well-being, reflect on the current discussions regarding the impact of Modern Treaties on Indigenous livelihoods, and consider how this might be evaluated. As a case study, we discuss the Nisga’a Lisims Government (NLG) experience in developing a method to measure well-being in a Modern Treaty context. As illustrated in the NLG example, research that is participatory and takes on a mixed quantitative-qualitative design may provide credible, generalizable findings, and a respectful and responsible approach to measure aspects of Indigenous well-being.

## Conceptualizing Indigenous Well-Being

### Refocusing on Well-Being in the Indigenous Context

There have been increasing efforts by academics, as well as among Modern Treaty Governments, to develop well-being measures that reflect the commonalities and specificities of Indigenous communities and Nations in Canada (Walter and Andersen 2013). The literature and examples of communities developing well-being frameworks or describing well-being over long periods of time are scarce. Often, examples of this work emerge in the context of small-scale or community-focused research. Other times, research results from major projects or development initiatives dictate objectives (e.g., impact benefit agreements, or academic studies). Rarely do more comprehensive and longer-term planned well-being frameworks consider how to assess, carry out and evaluate the well-being experiences of Indigenous communities in urban and remote contexts over longer periods of time in relation to changing sociocultural, political and environmental contexts. This paper therefore seeks to add to this literature through a case study showcasing a longer-term approach to measuring characteristics of well-being developed and implemented by a self-governing Indigenous Nation in western Canada.

Three examples connect the ways of reflecting local Indigenous values and perspectives and well-being outcomes. In one study, the Arctic Council commissioned an Arctic Human Development Report developing social indicators for monitoring trends in human development and established a comprehensive knowledge base for the Arctic Council’s Sustainable Development Program (AHDR 2004: 15 *in* Nymand Larsen et al. 2014: 15). Barrington-Leigh and Sloman’s (2016) work on life satisfaction among self-identifying Indigenous peoples in the Canadian Prairies is an academic-led initiative focusing on the comparison of observable attributes and conditions that might lead to changes in experienced well-being among Indigenous peoples in Canada (Barrington-Leigh and Sloman 2016). They demonstrate that using a single, well-specified measure of individuals’ self-reported overall satisfaction with life to assess well-being can both enable participants and communities to self-define their own criteria for prosperous and fulfilling lives while enabling comparisons between different societal groups (Barrington-Leigh and Sloman 2016).^5^ As well, Parlee and O’Neil (2007) developed a well-being assessment framework based on the values, knowledge, and institutions that underpin the “Dene way of life” with the Denesoline community of the Lutsel K’e Dene First Nation. This assessment allowed the community to articulate their priorities and is an example of a participatory method Indigenous communities can use to measure their development and well-being over time.

These examples provide a glimpse at the changing ways Indigenous communities, academics and concerned stakeholders are rethinking well-being as a community led priority. These studies also demonstrate that the methods used can be sustainable over longer periods of time, thus avoiding a more reactionary approach to measuring well-being by responding to industry, major project development, funding opportunity or academic interest. However, much of the current academic literature relates Indigenous well-being to social determinants of health. This is problematic as there is no definitive list or standard definition of social determinants or well-being for Indigenous peoples. This may, in part, explain the disconnected manner in which research and the objectives employed to define well-being have been examined by multiple actors, including industry, government, academy, First Nations, and others.

### Well-Being in the Indigenous Context; the Importance of Participatory Research

Indigenous well-being has broadly been described as holistic, multidimensional, and based on community-centred experiences and Indigenous epistemology. It has been associated to individual, systemic, and institutional factors, most notably, colonialism (Greenwood et al. 2018; Richmond and Cook 2016). For example, the medicine wheel approach, which is used in the First Nations Holistic Policy and Planning Model, the Integrated Life Course, and Social Determinants Model of Aboriginal Health, exemplifies the concept of Indigenous well-being as a holistic and integrated approach to health. It echoes the Misipawistik Cree notion of *E-Opinitowak*, meaning the act of “lifting ourselves up, empowering the community and promoting self-reliance” (Wien et al. 2019:11). These approaches broadly depict the holistic, integrated approach to Indigenous well-being whereby one balances aspects of the spiritual, physical, mental, and emotional components of one’s life.

However, applying a holistic framework to comprehensively assess the impacts of Modern Treaties on Indigenous well-being is challenging. Integrating differing cultural and traditional knowledge to ensure the meaningful and relevant interpretation of results is also difficult given the complex interplay of determinants that underlie the diversity of Indigenous well-being experiences. These may also be quite complex, if not impossible, to report and monitor through conventional statistical instruments. As argued by Drawson, Mushquash, & Mushquash, “data analytic methods may lack the sophistication to account for complex relationships between variables” (Drawson et al. 2017:22). Given the limitations of the existing data collected by federal departments and agencies, developing indicators exclusively based on quantitative measures may be ineffectual on a longitudinal scale to describe well-being. These quantitative measures may also be unsuitable to compare Indigenous and non-Indigenous groups, inter and intra Indigenous communities, and experiences of historic and Modern Treaty holders.

The literature on community based participatory research provides additional insights on the ways in which the decision-making powers over the data collection process affect its outcomes, as it highlights “the mutual benefits of bi-directional research capacity and co-learning for those involved” (Castleden et al. 2016: 160). Considered as an essential means of effectively addressing socioeconomic and health disparities, this literature draws attention to positive outcomes generated by “the process of sharing expertise, decision-making, and ownership through equitable involvement of partners in all phases of the research from inception through to implementation and dissemination” of results (Ritchie et al. 2013: 184). In so doing, as explained by Kyoon-Achan et al. (2018: 1036), this approach “provides the opportunity to engage communities for sustainable change” by enhancing their ability to identify and address the determinants of Indigenous well-being.

While qualitative studies provide rich contextual information and detailed explanations, the substantial costs associated with these studies, and the difficulty in generalising their findings, often restrict their application in large-scale, longitudinal inquiries. As such, strictly qualitative research has not often been employed to look at the impacts of modern treaties on Indigenous well-being.^6^ In contrast, mixed quantitative-qualitative designs provide the standardization of a quantitative approach with the interpretative benefits of qualitative design, which can lead “to both credible, generalizable findings and accurate measurement of phenomena” (Drawson et al. 2017:21). We suggest such an approach is beneficial when engaging with Indigenous ways of knowing to develop “relevant, reciprocal, respectful, and responsible research” (Peltier 2018:1) by, with, and for Indigenous peoples (Kolahdooz et al. 2015; Levac et al. 2018).

## Federal Government Initiatives to Measure Well-Being in Canada

The evolution of the Canadian government’s approach to measuring the social and economic well-being of Indigenous peoples begins with early efforts to increase public awareness of persistent inequalities between Indigenous and non-Indigenous peoples, and to inform public policy discussions about the efficacy of public expenditures (Cooke et al. 2004:48; see also Kenny 2014; O’Sullivan 2011; Steffler 2016). To accomplish this the government relied on two indexes, the Human Development Index and the Community Well-being Index, which were used to measure and evaluate the socio-economic well-being of Indigenous peoples.

### The Human Development Index

In 1999, Aboriginal Affairs and Northern Development Canada (AANDC, now CIRNAC) created the Registered Indian Human Development Index (Registered Indian HDI) in an attempt to systematically describe the changes in the relative well-being of the Registered Indian population and other Canadians in a single, easily understandable measure modelled after the United Nations Development Program’s Human Development Index, with scores ranging from 0 (lowest level of well-being) to 1 (highest) (Cooke et al. 2007). Using census data produced by Statistics Canada, the Registered Indian HDI offered national and regional measures of educational attainment, income and life expectancy.^7^ The HDI attempts to capture the conditions that are needed for people to lead long and healthy lives. However, as argued by Cooke et al. (2004), while this index reveals important and continuing differences across these dimensions between Registered Indians and other Canadians, it does not fully capture well-being or expose community-level dynamics, especially for Indigenous populations.

### The Community Well-Being Index

In 2001, Indian and Northern Affairs Canada (INAC, now CIRNAC) created the Community Well-Being Index (CWB) as a complement to the Registered Indian HDI. In Canada, the CWB remains the only tool for measuring the socio-economic well-being of individual First Nation, Inuit, and non-Indigenous communities across Canada (O’Sullivan 2011; O’Sullivan and McHardy 2004).^8^ The CWB consists of a numerical score ranging from zero to 100 which is calculated by adding four equally-weighted components that encompass the following indicators: 1) total income per capita, 2) education (high school and post-secondary graduation), 3) housing (overcrowding and need for major repairs), and 4) labour force (participation and employment). Well-being scores, based on the CWB, have been calculated from data collected by Statistics Canada through a mandatory long-form questionnaire, the National Population Census, every five years starting in 1981, except in 2011, when the Census was replaced by the National Household Survey (Smylie and Firestone 2015).

The CWB index offers a simple representation of a complex and multifaceted phenomenon in a way that facilitates the evaluation and comparison of trends for Indigenous and non-Indigenous communities over time. As such, academics and others have used the CWB index on its own, or alongside other primary and secondary data sources, to examine and illustrate socio-economic disparities between Indigenous and non-Indigenous Canadians between 1981 and 2016 (Baron et al. 2019; Bougie et al. 2015; Cooke and O’Sullivan 2015; Oliver et al. 2016; Penney et al. 2012).

In 2013, AANDC (now CIRNAC) used the CWB to explore the impacts of treaties on First Nations (Guimond et al. 2013). AANDC’s analysis revealed that, on average, both Modern Treaty and non-Treaty First Nations displayed higher well-being levels than historic treaty First Nations.^9^ The well-being of Modern Treaty First Nations was shown to improve twice as fast as historic treaty First Nations between 1981 and 2006 (Guimond et al. 2013). However, the authors recommended interpreting their findings with caution as it appeared “difficult to distinguish the impact of treaties on well-being from the impact of regional factor[s] [with] better-off First Nations more likely to engage in and successfully conclude modern treaty negotiations” (Guimond et al. 2013:3).^10^ It is important to place Guimond et al. (2013) and others’ work in context. For instance, there are different types of historic treaties settled differently (Russell 2017). The implementation of the spirit, goals and intent of treaties varies as well. Such contextual factors inevitably affect the extent to which regionalism matters, and how treaties differently impact on the well-being of Indigenous beneficiaries.

Although the CWB index may provide a means of assessing how the process and outcomes of Modern Treaty implementation affects the lives of Indigenous communities, it is neither comprehensive nor culturally adapted. Fundamentally, the CWB was neither designed nor intended to fully represent the complex intersections and interrelations of determinants that impact the health of Indigenous persons, communities, or Nations across Canada, nor was it conceived as a tool for assessing the impacts of modern or historic treaties (Wilk et al. 2018). This was highlighted in the 2018 Auditor General of Canada’s report, which found that the CWB did not adequately measure well-being as it excludes important well-being dimensions, including health, land-based relationships, language, and cultural vitality (Canada Parliament. Office of the Auditor General 2018).^11^

The CWB is unable to disaggregate scores by age and sex, and excludes Métis people and Indigenous peoples living off reserve (Wilk et al. 2018). While the components of the CWB (e.g., income, education, housing, and labour force activity) can be examined separately through an interactive, online platform, CWB scores are only available for communities with a population of at least 65, and for sub-component scores for those with at least 40 households and 250 individuals (O’Sullivan 2011). The comparability of community scores can be further limited by the inconsistent participation or the lack of participation, including the non-participation, of Indigenous communities in national censuses(Penney et al. 2012).^12^

### The Difficulties and Opportunities in Measuring Indigenous Well-Being in Canada

One of the main factors limiting the ability of governments and researchers to comprehensively reflect Indigenous well-being experiences and to assess treaty impacts is the nature and reliability of the data collected for administrative purposes. Data gathered through program or policy evaluations are often designed for reporting, compliance, and accountability requirements rather than to assess their outcomes or effects on Indigenous living conditions. Steffler (2016) states that the information “collected for operational and/or legislative requirements or reporting requirements under the terms and conditions of funding agreements” (Steffler 2016:152–53), otherwise known as administrative data, tends to lack metadata (data dictionaries and methodological frameworks), standard demographic data fields, or methods to identify particular Indigenous people whom may utilize a specific program or service. Strict privacy rules and conditions further restrict the access and use of these data sets, while the narrowly defined objectives they were intended to serve also limit their usefulness for well-being assessment. Additionally, most data sources in Canada either lack an Indigenous *identifier,* or a way to understand the connection to one’s culture or homeland.^13^ Insufficient Indigenous representation in the sampling methodology may also prevent the production of reliable disaggregated estimates, as with the data produced by Statistics Canada (Steffler 2016). Moreover, while Census data are dependable, these are only collected every five years.^14^

Our inability to determine whether, under what conditions, and to what extent Modern Treaties improve the living conditions of Indigenous signatories compelled the Federal government to examine potentially suitable methodologies and indicators to this end (Kenny 2014). Following a review of practices for measuring and assessing the impacts of Modern Treaties on Indigenous well-being for AADNC, Kenny (2014) provides a departmental guide that identifies several issues that need to be addressed. Paraphrased here, these include: 1) forming collaborative partnerships in the development of relevant and culturally-appropriate indicators; 2) funding and training for the capacity-building of signatory communities; 3) clearly articulating the goal of the measurement process and outcomes for all involved parties measuring the impact of Modern Treaties; 4) developing indicators that take into account the uniqueness of individual communities; and 5) conducting a baseline assessment to evaluate well-being prior to treaty negotiations.^15^

A subsequent report by the Canada Standing Committee on Indigenous and Northern Affairs (2018), which mirrors Kenny’s (2014) earlier work, presents several recommendations to ensure that Modern Treaties benefit Indigenous communities. These include that Canada: 1) “adopt a holistic and flexible approach rooted in the recognition of rights for the resolution of comprehensive claims”, which recognizes that “a one-size-fits-all policy for the country will not work”, and that land claims agreements are living documents; 2) support “Indigenous community-led data collection, with a focus on using this data to improve and accelerate the implementation of comprehensive land claim agreements, and to hold government accountable for implementation of these agreements”; 3) develop a tracking system to ensure that the commitments made by the federal government are “clearly documented, the progress regularly reviewed, and promptly implemented; and that an independent office be created to monitor implementation” (Canada Standing Committee on Indigenous and Northern Affairs 2018:3–7). A more comprehensive evaluation and monitoring methodology is expected to be developed as data from the Modern Treaty Management Environment, together with a performance measurement framework, becomes available and by further incorporating the perspectives and experiences of Indigenous peoples with the implementation of modern treaties and self-government agreements to the reporting process (CIRNAC 2019). Despite sustained interest in addressing better techniques to evaluate the impacts of Modern Treaties and focused conversations between CIRNAC and Indigenous Governments, results that resonate for many Modern Treaty partners have been slow to materialize.^16^

## Well-Being Indicators and Self-Government

Culturally and contextually relevant well-being indicators may provide Indigenous governments with the capacity to efficiently respond to local priorities and concerns (Smith 2016). Theoretically, if citizens of self-governments are doing well and thriving – a Modern Treaty is *working* for the people as it should. This connection between well-being indicators and governance is rooted in the way a government’s performance is measured.

Effective governance, whether for small groups or large nations, means being capable of “future-oriented planning, problem solving, evaluating outcomes, developing strategies and taking remedial action” (Smith 2016:124). Having these capabilities requires demographic facts and contextual knowledge of the strengths, assets, resources, and expertise a nation, community, or organisation already has and can bring to bear. Effective governance also requires knowledge of a community’s existing infrastructure, technology, funding sources and constituent base, including understanding how organizations engender trust or create social norms (Putnam 1993; Smith 2016).

We see data and data governance as key to effective Modern Treaty governance. Effectively measuring the performance of a Modern Treaty must account for regional diversity and history, as well as identifying well defined indicators that may be used to track change over time. Articulating the state of well-being for a Modern Treaty Nation will require incorporating multiple perspectives and transparency in defining indicators, particularly when it comes to determining what to measure and how to measure it. As argued by Yap and Yu (2016), self-determination overarches all aspects of Indigenous well-being, which encompasses “autonomy over one’s life and how individuals choose to live their life, [and is] also about people’s autonomy over the decisions and responsibility to care for and manage their country and land as part of their existing and enduring well-being” (2016:317).

Data governance, defined by Smith (2016) as “…the power and authority to make rules and decisions about the design, interpretation, validation, ownership, access to and use of data” (2016:119) is currently limited for Indigenous governments because they have a restricted access to existing data produced and collected by other governments or parties. Indigenous governments and organizations struggle to meaningfully partake in the definition of a well-being framework given their lack of control of existing data. Inadequate financial and human resources pose additional challenges to Indigenous data governance. However, a number of Indigenous groups and their governing bodies are choosing to produce, interpret, and manage their own information systems and databases (Smith 2016). NLG’s research is one example of an Indigenous government taking a more proactive role to capture relevant baseline data detailing the circumstances of Nisga’a citizen’s lives, particularly as it relates to well-being.

## The Nisga’a Nation Household Survey

The Nisga’a Nation is located on the northwest coast of British Columbia (BC), in the Nass River Valley, northwest of Terrace (see Map 1). As stated on the Nisga’a Lisims Government (NLG) website, “the Nisga’a people have lived in the Nass River Valley since before recorded time,”^17^ guided by Ayuukhl Nisga’a.^18^ On May 11th, 2000, the Nisga’a Final Agreement came into effect, and Canada and British Columbia formally recognized Nisga’a ownership of 2000 km^2^ of Nisga’a lands, as well as the resources on or under those lands.^19^ The Nisga’a Nation is comprised of both national and local governments: NLG is responsible for governance of the Nisga’a Nation as a whole, and each of the four Nisga’a Villages — Gitlaxt’aamiks, Gingolx, Gitwinksihlkw, and Laxgalts’ap —exercises functions related to the village and its residents.^20^

**Fig. 1:**
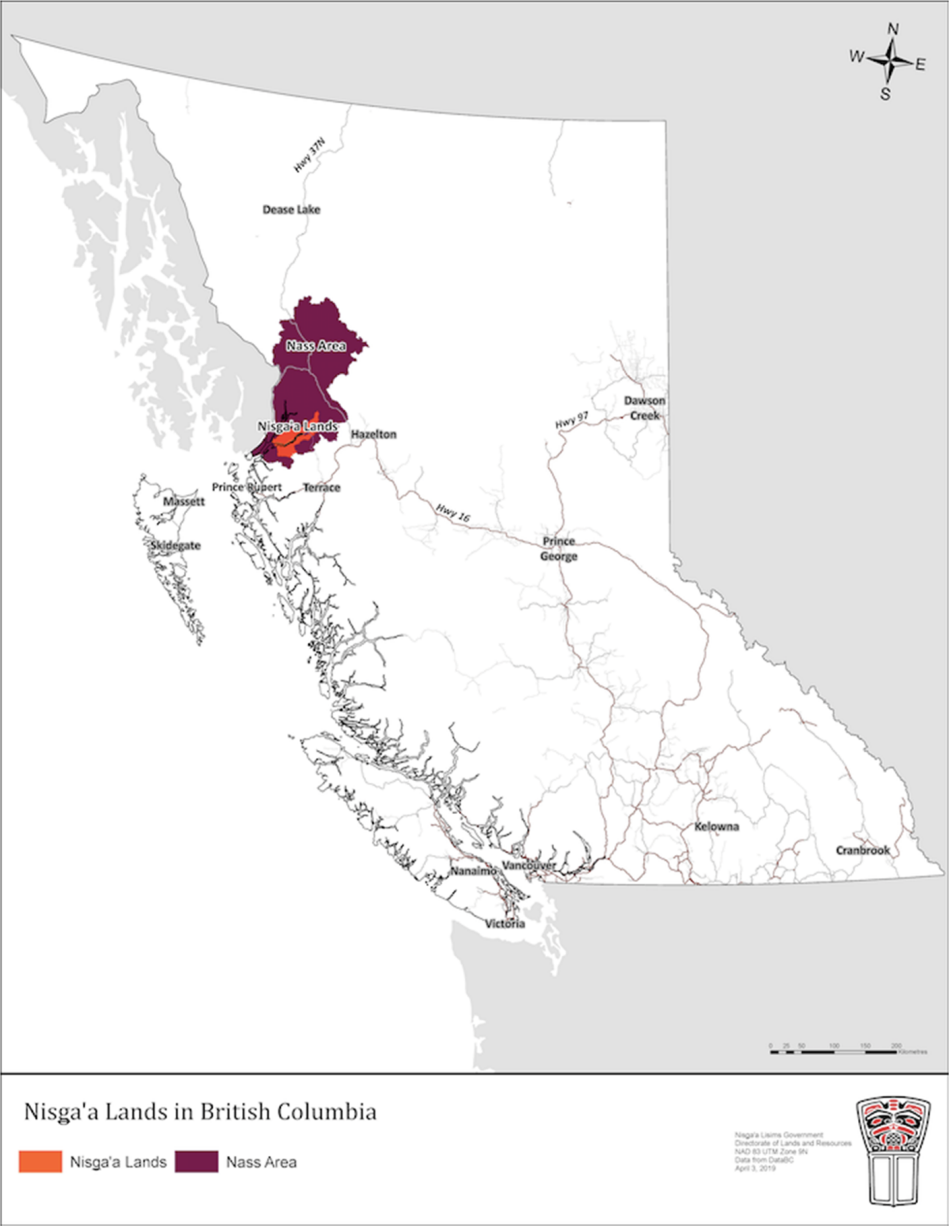
*British Columbia and Nisga’a Lands in the Nass Valley*. Source: Nisga’a Lisims Government Custom Map

As of May 2019, 1492 Nisga’a citizens aged 15 and older live on Nisga’a lands, and an additional 4408 live off Nisga’a lands in Terrace, Prince Rupert, the Lower Mainland/Vancouver Island, or elsewhere in BC (Perry 2020:9). According to NLG’s 2020 Labour Market Study, the population aged 15 and older is projected to increase from 5900 in 2019 to 6656 by 2029 (Perry 2020:10). In terms of the labour market, the Nisga’a Nation consistently displays higher unemployment rates and lower employment rates than the rest of the province, but participation rates have remained stable since 1991 (Perry 2020:16). Natural resource extraction has been and continues to be a driver of economic development for the Nisga’a Nation. In 2018, LNG Canada announced a positive Final Investment Decision (FID) for the development of their Kitimat-based LNG export facility, which is expected to create new jobs and spur increased economic activity in the region (Perry 2020:6). (Fig 1).

The Nisga’a Nation, as represented by NLG, provides a case study of a culturally adapted method of acquiring baseline data using a mixed quantitative and qualitative approach. This approach assists NLG in assessing the impacts of the Nisga’a Final Agreement, a Modern Treaty and a land claims agreement within the meaning of sections 25 and 35 of the Constitution Act, 1982.^21^ NLG’s work focuses on administering a household survey in an attempt to garner feedback from Nisga’a citizens. The survey, which included the administration of a questionnaire, was a pilot study conducted over the fiscal 2018/2019 year. Currently, NLG’s administration is considering whether to administer the survey in five years’ time (2022/2023), pending feedback and approval from NLG’s Executive. In the initial development and design of the survey, an initiative of NLG’s ‘Quality of Life’ (QoL) department,^22^ administering a survey at intercensal periods (occurring between censuses) was considered.^23^ The intercensal survey would allow NLG to collect longitudinal data that could generate quality estimates and inferences about the Nisga’a populations in the four Nisga’a Villages in the Nass Valley, as well as other major urban areas in the province of British Columbia where Nisga’a reside. QoL staff were responsible for the implementation of the pilot survey, including questionnaire administration, and the subsequent data analysis.

While non-Nisga’a statistical agencies already collect information to produce reports about Nisga’a (and other Indigenous peoples), NLG undertook its own household survey to supplement the other information being gathered and analyzed. The combined information resulted in high quality statistical data, and data that qualitatively expands the understandings provided by quantitative information. The survey approach was driven by a need to communicate community-level data to Nisga’a Village members, administrators and elected officials to support data informed decision-making about programs and services. The NLG survey confirmed NLG’s concerns regarding conventional survey methods as conventional survey results were found to underrepresent actual circumstances for the Nisga’a. Previously collected conventional survey material has had limited use in helping to articulate and describe the unique circumstances and the quality of life for the Nisga’a Nation.

To this end, quantitative data were collected through the responses given by Nisga’a citizens. The questionnaire also collected qualitative responses that expanded on the quantitative questions. The questionnaire was used to collect: (1) demographic information, (2) household food security, (3) transportation, (4) infrastructure and assets, (5) housing, (6) water services, infrastructure and waste management, (7) traditions and cultural practices, (8) skills and training needs, (9) activities of daily living, and (10) monthly income, expenditures, and agricultural productivity (supplement) information. See Table 1 for examples of some of the questions included in the NLG household survey.

**Table 1:** NLG Household Survey: Question Examples

Question #	Question
1.1	**Please provide the following information about your household (starting with the household** **head):**
	• Age
	• Gender
	• Marital Status
	• Highest Education
	• Employment Status
	• Occupation
2.1	**Is it easy to access food in your community? Explain:**
2.2	**What challenges are you faced with in accessing food?**
	1. Distance to shops
	2. Lack of funds to travel
	3. Lack of transportation
	4. No local shops (i.e., in the Nass Valley)
	5. I don’t have an issue
	6. Rising cost of food
	7. Other (list)
5.3	**Dwelling type**
	1. Single-detached house
	2. Apartment building (five or more stories)
	3. Semi-detached house
	4. Apartment / duplex / row house
	5. Movable dwelling (i.e. trailer, mobile home, camper)
	6. Other
5.5	**Do you have any structural damage or concerns with your house?**
	1. Yes
	2. No
	3. Unsure
7.7	**How well does your family know Nisga’a art and culture such as weaving, marking ceremonial regalia, ornaments, carving, or jewellery making?**
	1. Very well
	2. Pretty good
	3. Somewhat (partial understanding)
	4. Very limited
	5. Not at all
	6. Decline to answer
7.9.1	**Have you been to a Nisga’a cultural event in the last year?**
	1. Yes
	2. No
7.11	**How well do you know the Nisga’a language?**
	1. Not at all
	2. A few words and phrases, such as greetings and giving thanks
	3. I can understand what is said but I cannot speak
	4. I can converse in the language when others who are fluent speak with me
	5. I am fully fluent in all aspects of the language
8.2	**In the last year, have you undertaken any training?**
	1. Yes
	2. No

Although time consuming to administer, NLG created a questionnaire that allowed them to go beyond numbers to the stories that make numbers more meaningful and useful for planning. This approach increased NLG’s ability to more effectively respond as a government. While the quantitative questions used multiple choice, benchmarking, rating scale, and Likert-like scale types of questions, space was provided for open-ended descriptive text as well. Qualitative responses were recorded as respondents could further expand upon their experiences when providing answers to the themes and questions asked, thus allowing for elaboration beyond the structured questions found on the questionnaire.^24^ In instances where literacy was an issue or Elders required assistance, NLG’s research assistants worked closely with interviewees to fill in the responses.^25^

Some specific challenges noted during the design stage included the time required to educate interviewers on mixed-method approaches, including the need to methodically and neatly write out qualitative responses. Based on lessons learned from the pilot project, to be used for future surveys, NLG would engage in more structured training that would occur much earlier in the process; the training would also focus on the varied techniques to hand record quantitative and qualitative information.^26^ Furthermore, instead of hiring interviewers from each Village/Urban Locale and training these interviewers in cohorts, NLG would hire across the Nation and train interviewers within a two to three week course. This approach would likely improve the capacity training required and could save time and costs.

The feedback received from senior officials and Elders was paramount for the questionnaire’s finalization. To this end, the questionnaire was piloted with several Nisga’a Elders’ groups in Terrace and Prince Rupert prior to its administration. This process was beneficial for refining specific wording for questions, and for articulating the extent and purpose of the research. The questionnaire was then administered to a random sample of individuals from the Nisga’a population living in urban areas. NLG’s survey methodology combined the use of convenience and random sampling to collect information for Nisga’a citizens living in urban locales (off Nisga’a Lands), as well as a census of those living in Nisga’a communities.

Non-response bias was noted (village/urban and over/under representation) by comparing grouped age cohorts to the actual population sizes (Perry et al. 2020). Younger adults (both females and males) in the villages (18–29) were underrepresented. This reflects the need to build better trust relationships between government and younger adults, although a number of factors such as work, schooling, and tending to family obligations equally impacted the low response rate. In the urban centers, over/under representation and non-response bias was nuanced and displayed variation. Noticeably, males aged 30–39 were underrepresented; with survey administrators noting that these respondents were working and had little time to take the survey.

NLG trained and hired local investigators from the four Nisga’a Villages and three urban centers for a total of 28 hires. Canvassers were trained by the QoL manager, who is experienced in research design and implementation, and were given field experience practice through mock interviews and practical skills training in research methods prior to going door to door to conduct interviews. In each community canvassers were supported, and supported one another, through morning meetings used to establish routes and sub-sections to canvass within the communities. Following the morning meeting, the canvassers went out in pairs. There were also daily late afternoon and evening debriefing sessions to ensure that canvassers were supported and felt safe to voice any concerns encountered during field research. In some instances, (the urban case) – higher turnover among staff led to hiring older and more mature Nisga’a representatives/interviewers to ensure accountability among younger less experienced staff. This was done to ensure quality responses and provided confidence that the work was carried out with a level of rigour desired by NLG administrators.

A survey of 30.4% of the Nisga’a population overall (see Table 2) was conducted throughout the province of British Columbia. Non-response rates for households demonstrate patterns that are similar to current non-response rates for similar types of household surveys using similar survey methods^27^ (see Tables 3 and 4). The NLG survey was as effective in obtaining responses as Statistics Canada and other surveys administered on Nisga’a Lands by third parties. Additionally, the NLG survey added value by obtaining qualitative data that was used to contextualize the quantitative information collected.

**Table 2:** Nisga’a Survey and Census Numbers

Village	Total no. of individuals 18+ (from population list)	Total no. of individuals 18+ where info was collected (full survey)	% of coverage (18+)	Random selection sample size (18+) 95% CL^1^ 10% MoE^2^ 50% RD^3^	No. of individuals surveyed from the random list (18+)	% of coverage for random sample (18+)
Prince Rupert	1115	203	18.2%	89	25	28.1%
Terrace	917	166	18.1%	88	39	44.3%
Vancouver	840	144	17.1%	87	15	17.2%
Totals (urban centres)	2872	513	17.9%	264	79	29.9%
Gingolx	301	149	49.5%			
Gitwinksihlkw	147	101	68.7%			
Gitlaxt’aamiks	578	304	52.6%			
Laxgalts’ap	394	219	55.6%			
Totals (villages)	1420	773	54.4%			
Overall Totals	**4292**	**1286**	**30.0%**			

^1^confidence interval

^2^margin of error

^3^response distribution

**Table 3:** Non-response rate for Nisga’a living in urban centres

	Prince Rupert ^1,3^	Terrace^1^	Metro Vancouver^2,3,4^	OVERALL TOTALS
Sample (HH^5^)	494	501	494	1489
Actual Interviews (HH)	86	81	69	236
RESPONSE TOTALS (HH)	86	81	69	236
RESPONSE TOTALS (% of HH)	17.4%	16.2%	14.0%	15.9%
Refusal (HH)	20	15	22	57
Refusal (% of sample)	4.0%	3.0%	4.5%	3.8%
No answer (HH)	34	18	78	130
No answer (% of sample)	6.9%	3.6%	15.8%	8.7%
Recently moved (HH)	16	26	12	54
Recently moved (% of sample)	3.2%	5.2%	2.4%	3.6%
NON-RESPONSE TOTALS (HH)	70	59	112	241
NON-RESPONSE TOTALS (% of HH)	14.2%	11.8%	22.7%	16.2%

^1^4 attempts made at HHs with no answer

^2^1 to 2 attempts made at HHs with no answer

^3^Unsure of total number of HH (sample HH estimate calculation = 3 members per HH/ total population)

^4^There are 2 HH with 2 people in each HH from the Vancouver HH surveys, one from Victoria, and one from the Southern Gulf Islands

^5^HH = Households

^6^The totals displayed will not equal 100% as we used a hybrid sampling method, random sample and sample of convenience, and not a complete census of the population

**Table 4:** Non-response rate for Nisga’a living in Nisga’a villages/communities

	New Aiyansh / Gitlaxt’aamiks^1^	Gitwinkshilkw^2^	Laxgalts’ap^3^	Gingolx^3^	OVERALL TOTALS
Sample (HH^4^)	264	42	135	125	566
Actual Interviews (HH)	125	35	94	67	321
RESPONSE TOTALS (HH)	125	35	94	67	321
RESPONSE TOTALS (% of HH)	47.3%	83.3%	69.6%	53.6%	56.7%
Refusal (HH)	50	3	16	15	84
Refusal (% of HH)	18.9%	7.1%	11.9%	12.0%	14.8%
No answer^4^ (HH)	70	3	15	35	123
No answer (% of HH)	26.5%	7.1%	11.1%	28.0%	21.7%
Recently moved (HH)	19	1	10	8	38
Recently moved (% of HH)	7.2%	2.4%	7.4%	6.4%	6.7%
NON-RESPONSE TOTALS (HH)	139	7	41	58	245
NON-RESPONSE TOTALS (% of HH)	52.7%	16.7%	30.4%	46.4%	43.3%

^1^2 attempts made at HHs with no answer

^2^4 attempts made at HHs with no answer

^3^3 attempts made at HHs with no answer

^4^HH = Households

^5^Some of the individuals included in non-contact (no answer) had missed appointments

### Analyzing Nisga’a Survey Results

One of the challenges encountered when analyzing the data, due to the nature and length of the questionnaire administration, was the length of time to analyze the very rich and detailed responses given both in terms of quantitative and qualitative data offered. Additionally, while community leaders and citizens pressed the QoL Department for more immediate results, generating the tables and graphs of results took longer than expected as the nature of the questionnaire and the time required to complete the analysis was increased given the amount of data collected.

The resulting compiled data has been used to verify intercensal estimates between official census dates and known data, as well as to infer estimates for the wider Nisga’a population. From the results, infographics and community profiles were compiled for specific Nisga’a communities as a means of deepening the conversation about the social realities of each community. Subsequent feedback from community members further helped NLG in understanding current and/or future issues that the respondents were, or will be, facing. For instance, previously there was an inability to capture and analyse the dynamics and circumstances of Indigenous peoples or populations who live in geographically diverse places. Some factors related to this dynamic include relocating for services, connection to culture, one’s heritage or cultural diversity, lifestyle preferences and choices, politics, and factors affecting the concept of *home* and connection to a homeland. The opportunity to undertake and administer a household survey unique to the context of the Nisga’a Nation allowed NLG to ask more specific questions about the cultural context and everyday realities of people living on and off Nisga’a lands, thus gaining a better understanding of dynamics and circumstances particular to the Nisga’a. Current conventional methods, which may not fully involve local community members as interviewers, may not fully capture important social dynamics that this mixed methods approach captured. NLG’s interviewers were able to unpack and find nuance within many of the questions asked.

The QoL data has been used to produce several reports on well-being, on demographic trends, labour force characteristics, food security, and housing, infrastructure, and water issues.^28^ The survey is also used to track changes in cultural practices on and off Nisga’a Lands. In some instances, the questions asked aligned to certain existing surveys administered by Statistics Canada. The associated analysis and reports on those themes and questions asked in the survey will be used to examine how NLG’s questions compare to other existing questions used on other statistical instruments. This was done intentionally to allow for comparison of our findings and population estimates to that of other surveys.

NLG’s example highlights a hybrid approach to survey data collection and methods and also demonstrates how collecting qualitative and quantitative survey information can be accomplished in partnership with interested stakeholders. Statistics Canada and CIRNAC played a role, albeit limited, in offering technical support to NLG’s questions related to sampling. For example, technical supports were offered to refine the sampling methodology employed by NLG’s administrators. Statistics Canada and the Modern Treaty Implementation Office (MTIO) provided feedback on the sampling approach employed by NLG, as well as the questionnaire layout, and design. Additionally, they offered feedback on labour force questions.^29^ Further external support from health statisticians would have been required to examine how additional data sets might complement and enrich the information drawn from the QoL Survey. In particular, NLG was interested in accessing health data that would show the link between industry impacts and health concerns such as chronic diseases and cancer rates.

Presently NLG is reflecting on how to incorporate different epistemologies into a future survey, as well as how the data from the survey can be shared. NLG’s investigators stress the importance of placing primary data collection strategies and methods back with those who best understand the context, culture, and questions relevant to ask of the people under study. This includes resourcing, capacity sharing, and time that is required between the Canadian government (Statistics Canada) and Indigenous governments to fully explore how new methods may be undertaken more collaboratively. More to the point, there is a concern from Indigenous governments regarding the need to go beyond certain statistical approaches that privilege the interests of dominant governments and stakeholders. It is critical that more decentralized approaches to data collection methods are explored and made relevant and inclusive of the people under investigation (Chatterjee 1993; Cottrell 2010; Moreton-Robinson and Walter 2010; Walter and Andersen 2013). Having more accurate and localized data, and a community-centered approach enables a more accurate assessment of Modern Treaty implementation and provides insight into how knowledge is appreciated, understood, and generated.

## Opportunities beyond the Tensions

Developing culturally relevant methods for measuring well-being of self-governing Modern Treaty holders is an important step for generating valid data that may demonstrate whether, and under what conditions, such agreements might effectively reduce socio-economic disparities and improve the quality of life of Indigenous communities. However, the use of unsuitable assessment tools and misaligned priorities risk undermining, rather than empowering, Indigenous governments and organisations. Government funding, generally channelled through programs designed in compliance with mainstream (*Western*) notions of modernity and progress, may also promote culturally unsuitable goals and frameworks that contradict or compromise Indigenous values.

The survey methodology and questionnaire adopted by NLG showcase how a self-governing Indigenous Nation can take on its own research agenda and create a methodology for collecting primary data that works for its government and its citizens (Walter and Andersen 2013). Such an approach might be better supported by receiving technical assistance from Canadian agencies, particularly if there is recognition that the control and interpretation of the data should be directed by the people involved in the study. Self-governing First Nations are better placed to undertake their own primary research agenda given that many have established governance systems that support their capacity to undertake this work.^30^

Similar work associated with a collaborative federal fiscal policy development process (Nicol et al. 2019) suggests that the outcomes are better for all parties when federal, territorial/provincial, and Indigenous governments work together to close social and economic disparities between Indigenous peoples and non-Indigenous people. All parties learn from each other, build capacity and promote reconciliation during such shared efforts.

To work around the limits and opportunities of existing data sources there needs to be greater collaboration between provincial/territorial, federal, and Indigenous governments, as well as a strong commitment among public servants and Indigenous organisations and their representatives to find synergies and mobilise existing tools and data (for examples, see Elias et al. 2014). For instance, Statistics Canada offers Public Use Microdata Files, available online, that are accessible to organizations and individuals to customize and interpret data, and create new results (Morris 2016). Aboriginal Liaison Officers, hired by Statistics Canada, provide 500 h of free data supports to selected national Indigenous organizations that must, thereafter, continuously re-invest the finite resources they possess to sustain and update the statistical products they develop.^31^

Additionally, by neglecting to provide important explanatory contexts, reports published by Statistics Canada may present relevant information in ways that could be perceived by Indigenous people as insensitive, impractical to their needs, or even misleading. Consequently, these could appear to reinforce prevalent stereotypes rather than to highlight meaningful trends and disparities that could inform effective policy-making (Morris 2016). As an example, any report speaking to education in an Indigenous context would be misleading unless it spoke of the long-lasting impacts of colonialism and residential schools on educational attainment. Similarly, other indicators where disparities persist between Indigenous and non-Indigenous people (such as health or housing) need to be contextualized within settler-colonialism and systematic discrimination to be effectively understood and diagnosed. The capacity and resources to identify and extract relevant data, as well as the ability to translate it into practical knowledge and insightful diagnostics useful to Indigenous governments and organizations is an important step in self-determination.

Government and academe might also provide greater support to Indigenous governments through resourcing, collaboration, and capacity building initiatives which enable Indigenous governments to measure and evaluate well-being on their own terms, including through primary data collection. While improving the accessibility of data collected from Indigenous peoples and Treaty Nations that provincial/territorial and federal governments have gathered over the years is important, the dialogue needs to extend to how this data will be utilized and for what purpose.^32^ Data sharing and joint analysis would enhance the process and outcomes by developing a more transparent, consistent, and contextual way of measuring the implementation, as well as the impacts, of Modern Treaties on Indigenous well-being.

## Conclusion

Exploring whether and under what conditions Modern Treaties may improve the lives of First Nations, Inuit, and Métis peoples requires the development of more comprehensive and contextually adapted survey methods. Looking at previous efforts to measure Indigenous well-being, the authors raise several suggestions regarding the use of mixed methods and surveys to support effective governance, whether for small groups or large nations. Further, the way in which governance bodies define and measure well-being should not merely be considered as an expression of a society’s values and goals, but also through the influence it exercises on peoples’ daily lives through government policies (Cairney et al. 2017).

Well-being frameworks and indicators foster constructive public discussions over development priorities and objectives, and ultimately reinforce the legitimacy, capabilities, resources and accountability of governments (Taylor 2008). A firmly grounded theoretical and historical understanding of well-being, as well as the strategic use of both quantitative and qualitative methods, may reveal dependencies, synergies, trade-offs, potentials, scaling, and cross-cutting issues that may be used to guide public policies. These revelations may ultimately promote cooperation between different societal actors, groups, organizations, as well as governments. As argued by the New-Zealand government, the cross-cutting nature of many social issues means that social well-being indicators are not necessarily a tool for evaluating the effectiveness of specific government policies per se. Rather, these enable governments, civil society, and citizens to monitor trends across key dimensions, compare national well-being scores with other countries, support better-informed public debates, inform planning and decision-making processes, and identify areas where actions are needed (Ministry of Social Development 2016). Indeed, well-being indicators may help us assess how, and the extent to which Modern Treaties affect Indigenous lives. However, unless they are rooted in a meaningful context, with indicators reflective of Indigenous values and priorities, these may not adequately or effectively advance Indigenous well-being.

Careful consideration ought to be paid to how well-being frameworks and indicators are defined, as well as the scale and frequency at which data should be gathered and analysed. The data collection process and outcomes must be guided by Indigenous needs and priorities. We must also consider whether these satisfy the spirit and intent of the culturally-based systems of knowledge of Indigenous people, while supporting their planning, service-delivery, and development aspirations (Smith 2016). The limited availability of longitudinal data in sufficient quantity and quality, national laws and regulations that protect the privacy of Canadian citizens, and inherently polysemic conceptions and experiences of well-being make building a comprehensive, pan-Canadian evaluation framework of Indigenous well-being extremely challenging.

Moving forward, stakeholders involved in collecting information from Indigenous people must seek innovative practices for the development of inter-governmental partnerships and collaborations with academic researchers cognizant of historic and Modern Treaties. They must optimize existing resources and tools and unashamedly highlight limitations. Smylie and Firestone (2015) urge meaningful engagement with Indigenous peoples to govern and manage data that is collected from them to support their self-governance. We must also collectively work towards establishing relevant, consistent, and inclusive ways to support the objectives of Indigenous governments by adding relevant questions and indicators in source datasets across Canada or create new source data. As an example of such an effort, the Environmental Health Research Division of the First Nations and Inuit Health Branch (formerly Health Canada) engaged with Indigenous communities to assist them in implementing community-based research to improve health and well-being “by building and supporting their capacity to identify, understand and control impacts associated with projects, programs or policies implemented within their territories” (Kwiatkowski et al. 2009:57).^33^ This work has been supported by the inclusion of unique *identifiers*, which may have the capacity to speak more directly to the Indigenous context.^34^

Addressing more fundamental relations of power that underlie the way in which the Canadian government has historically attempted to measure and evaluate well-being, as well as looking for opportunities for collaborating with Indigenous Nations implementing Modern Treaties to determine better ways to think about well-being is critical. As an Indigenous government, NLG found it important to incorporate Nisga’a values and cultural laws into reporting practices to its citizenry, and as such found it important to hire local area residents to assist in this research. Indigenous governments and communities must take the lead in defining the criteria intended to assess whether, and in what ways, the implementation process and outcomes of Modern Treaties impact Indigenous lives. In the spirit of reconciliation, including Indigenous voices in the evaluation framework, data collection, data analysis, and interpretation of the results of these processes is of vital importance.

## Footnotes

1 Modern Treaties include CLCAs with self-government agreements and those without self-government agreements.

2 We use the term Indigenous as the preferred collective noun to refer to the original peoples of North America and their descendants, including First Nations, Inuit, and Métis Peoples. Where Aboriginal is used, it reflects the terminology used in the Constitution of Canada, specifically the *Constitution Act, 1982*, Section 35 (2), in addition to other federal documents and data products (Statistics Canada 2019c). Where possible, specific community names are used in addition to or instead of these collective nouns. In 2016, there were over 1.6 million Indigenous people in Canada (Statistics Canada 2018a). Of these, approximately 58.4% are First Nations (including both those who are registered under the *Indian Act* and those who are not), 35.1% are Métis, and 3.9% are Inuit (Statistics Canada 2018a).

3 In Canada, the Aboriginal identity of a person is based on “whether the person identified with the Aboriginal peoples of Canada”, as defined by the *Constitution Act, 1982*, Section 35 (2) (Statistics Canada 2019c).

4 See, *Beckman v. Little Salmon/Carmacks First Nation*, [Beckman 2010] 3 S.C.R. 103.

5 The life satisfaction question asked in the survey is: “Now a question about life satisfaction. On a scale of 1–10 where ONE means dissatisfied and TEN means satisfied, all things considered how satisfied are you with your life as a whole these days?” (Barrington-Leigh and Sloman 2016: 2).

6 There are a few notable exceptions, including Papillon’s (2008) study on the impact of the 1975 James Bay and Northern Quebec Agreement on the quality of life of the Crees of Eeyou Istchee and the Inuit of Nunavik, and Rodon’s (2018) study on the effects of land claims agreements on the development outcomes in the Canadian Arctic.

7 While the United Nations Development Programme (UNDP) uses per capita GDP of the population aged fifteen and over with income to account for the average individual income, the Registered Indian HDI uses the average income of the entire population, with and without income, from all sources as reported in the national census (Cooke et al. 2004:52).

8 As explained by O’Sullivan (2011) “the methodology of the Registered Indian HDI could not be replicated exactly for the CWB, since life expectancy estimates for small populations are inherently unreliable. Moreover, the relevance of labour market activities to well-being in Canada and the housing concerns known to exist in Aboriginal communities encouraged the inclusion of labour and housing indicators in the community-level measure” ( 2011:3). The CWB index does not provide scores for the Métis population since there are only eight Métis-designated settlement areas in Alberta, which are smaller geographic subdivisions than CSDs.

9 Historic treaties refer to the 70 treaties signed between 1701 and 1923 by the British Crown and Indigenous groups (Canada. Crown-Indigenous Relations and Northern Affairs Canada (CIRNAC) 2008).

10 Multiple intervening factors, such as location, natural resources and economic opportunities, leadership and good governance, safe communities and healthy families could have additionally influenced the apparent relationship between modern treaties and improved well-being (Guimond et al. 2013).

11 The Office of the Auditor General of Canada ( 2018) additionally recommended mobilizing “the volumes of available data from multiple sources” ( 2018:5) that could enrich the CWB in order to “issue a comprehensive report on the overall socioeconomic well-being of First Nations people on reserves” ( 2018:5).

12 It is also worth highlighting several additional limits. CWB scores were not calculated in 1986 since data on dwelling conditions were not collected in that year’s census (O’Sullivan 2011). The scores’ comparability is further impacted by the replacement of the Population Census by the National Household Survey in 2011. The subtle, yet noticeable, amendments to questions on education in the 2006 Census make longitudinal comparisons difficult as the amendments may produce fluctuations, or an illusion of stability, in well-being scores, or among the constituent parts (Bougie et al. 2015). Community boundaries may also change between censuses, with territories either increasing or decreasing due to administrative or political aggregations or divisions. In considering longitudinal enquiries, changes in research design and method impact on the comparability of the scores, particularly for small population counts (O’Sullivan 2011).

13 A unique value that is associated with an individual metadata record.

14 Similar challenges are experienced in other Commonwealth countries that share a common pattern of mainly British colonization, comparably high levels of overall human development, and that are home to Indigenous populations facing persistent inequalities (i.e. Australia, New Zealand and the United States) (Cooke et al. 2007; Mitrou et al. 2014). The Australian *Overcoming Indigenous Disadvantage* framework was developed to achieve “practical reconciliation” through the pursuit of statistical equality between the standard of living of Indigenous and other Australians in the areas of health, housing, education and employment (Taylor 2008). The report measures Indigenous well-being across several intersecting dimensions, drawing heavily on national census and survey data, and are used to determine whether policies and programs are achieving positive outcomes for Indigenous Australians. The Living Standards Framework (LSF) developed by the New Zealand Treasury similarly considers the collective impact of policies on intergenerational well-being, defined in relation to the notions of kaitiakitanga (stewardship of all our resources), manaakitanga (care for others), ōhanga (prosperity) and whanaungatanga (the connections between us) (Burton 2018). Born from the Treasury’s mandate to promote higher living standards for all New Zealanders, the LSF was designed as a tool for substantive, measurable change and introduced as part of the Tax Working Group, mandated to review and create a more “future-focused tax system”, especially as it relates to the potential taxation of Māori assets (O’Connell et al. 2018). The US Environmental Protection Agency (EPA) developed a human well-being index to empower and inform decision makers to proportionally weigh (or to assess in an equitable manner), the human health, socio-economic, environmental, and ecological factors that foster sustainability in a community at national, regional and local scales (Summers et al. 2017).

15 Kenny (2014) also notes that the AANDC focuses too much on indicators which measure outcomes from programs and services that are not necessarily relevant or useful to communities.

16 Conversations between the Modern Treaties Implementation Office (MTIO) and the Land Claims Agreements Coalition (LCAC) partners, as well as conversations at the Collaborative Fiscal Policy Development Process, have tended to focus on performance measurement and logic model development – including the selection of indicators that can be used to track change over time. The collaborative technical work between representatives of the Self-Governing Indigenous Governments (SGIGs) and the federal officials in defining and addressing appropriate indicators to use between stakeholders has been slow to produce results due to diversity of context and capacity among stakeholders. Subsequently, some Indigenous partners, who have both technical and/or financial capacities, have mobilized to refine specific methods for collecting primary data on housing and early childhood learning indicators amongst the SGIG group.

17 See, https://www.nisgaanation.ca/about-10

18 The Nisga’a Nation has a strong sense of culture and heritage, which is reflected in NLG’s system of governance and investment priorities. As stated on NLG’s website, “NLG is committed to protecting and promoting Nisga’a culture in all aspects of society”, and does so with the guidance of Ayuukhl Nisga’a, “an ancient and sophisticated code of laws, customs, and conduct.” As part of NLG, the Ayuukhl Nisga’a Department (AND) “protects, preserves, and promotes Nisga’a language, culture, and history” through a variety of initiatives and programs. See, https://www.nisgaanation.ca/about. Note that language revitalization is a priority for AND, given that between 2006 and 2016, the number of Nisga’a citizens whose mother tongue was Nisga’a decreased from 665 to 415 (Statistics Canada, Census 2019a, 2019b, 2020 & Statistics Canada 2018b: 97–555-XCB2006015, 98–314-XCB2011031, 98–400-X2016054).

19 The Nisga’a Final Agreement was signed in 1998 and came into effect in 2000. It is BC’s first Modern Treaty. See, https://www.nisgaanation.ca/about-accomplishments-and-benefits-nisgaa-treaty.

20 See, https://www.nisgaanation.ca/about-3.

21 See, https://www.nisgaanation.ca/treaty-documents

22 See, https://www.nisgaanation.ca/history-and-timeline;https://www.nisgaanation.ca/about-1

23 See, https://www.merriam-webster.com/dictionary/intercensal

24 Qualitative responses were hand-recorded by interviewers. These recordings often reflected personalized stories of family history, the day to day circumstances and challenges people faced, reflections about the impacts of residential schools, and other personalized stories. The commonalities of stories were noted and spoken of in general terms during subsequent participatory engagements to gain further feedback from community members regarding ways to improve the quality of life for citizens.

25 In a few instances, some Elders desired that the questions were communicated in the Nisga’a language. Research assistants that spoke fluent Nisga’a made special appointments with these Elders and assisted to orally translate the questions and record responses from these Elders.

26 If a further iteration of this work occurs – NLG will consider using electronic means such as tablets to collect information, in addition to hand recording qualitative responses. Retaining the option to hand record information is felt important given the comfort citizens conveyed by having one’s interviewer record information by this method.

27 Non-response rates for similar types of surveys average from 52.7% (Baruch and Holtom 2008) to 57% (Lindemann 2019). The suggested goal for response rates for research is response rates nearing 60% (Fincham 2008). However, Morton et al. (2012) suggest that response rates ranging from 5% to 54% may be acceptable.

28 See https://www.nisgaanation.ca/reports-and-graphics

29 NLG hired the professional supports of Big River Analytics in the conception and design of the household questionnaire, particularly on incorporating questions that would align to the Canadian census in order to verify results from the census. The questionnaire incorporated themes relevant to NLG’s QoL framework (https://www.nisgaanation.ca/about-1), with questions related to Nisga’a culture and context and the traditional harvesting cycles of working with the land. NLG received other academic advice and feedback on the questionnaire design from University of Northern British Columbia, Simon Fraser University, Thompson River University, Carleton University and Université Laval.

30 In recent decades, Indigenous self-government has advanced in the sense that the Crown has supported the legitimacy of these governments. The subsequent capacity of these governments to establish similar functions as federal, provincial, and territorial governments is increasing. The momentum described here hints at increased internal capacity by Self-Governing Indigenous Governments (SGIGs) in their pursuit of self-determination to advance control over service delivery, which includes determining better use of statistical data and the ability to take on primary data collection.

31 Outside of this paper’s scope but worth noting is the extent to which funded research and carrying out statistical analysis extends to Indigenous organizations. As an extension of federal, provincial or a territorial service, this support focuses the attention away from more directly backing modern-treaty signatories and Indigenous Governments in their endeavours.

32 The Ownership, Control, Access and Possession (OCAP™) principle advocated by the First Nations Information Governance Centre (2018) and the right to self-determination asserted by the United Nations Declaration on the Rights of Indigenous Peoples (2007) speaks directly to this point.

33 Other examples might include the inclusion of indicators that track the social and environmental impacts of industry, such as: monitoring water sources impacted by industry for quality and public safety, determining impacted natural resources used for important cultural activities, or benchmarking industry HR policies in hiring locally to best practices that exemplify better working relationships between companies and Indigenous governments and communities (For context see, Wilson et al. 2018).

34 See https://www.ngdc.noaa.gov/wiki/index.php/Data_Set_Identifiers_and_other_Unique_IDs
